# Supplementary material for: Scale invariance of BMP signaling gradients in zebrafish
Source: Sci Rep. 2019 Apr 1;9:5440. doi: 10.1038/s41598-019-41840-8 (PMC6443670; doi:10.1038/s41598-019-41840-8)
Supplement: Supplementary file 1 — Figure S1. The interpretation of how the curve of Hill equation (equation 1) changes with each parameter [file 41598_2019_41840_MOESM1_ESM.pdf]

Figure S1 - The interpretation of how the curve of Hill equation (equation 1) changes with each parameter

Scale invariance of BMP signaling gradients in zebrafish

Yan Huang[1], David Umulis[1,2,*]
[1]Agricultural and Biological Engineering
[2]Weldon School of Biomedical Engineering, Purdue University, West Lafayette, IN, 47907, USA.
[*]Authors for correspondence(dumulis@purdue.edu)

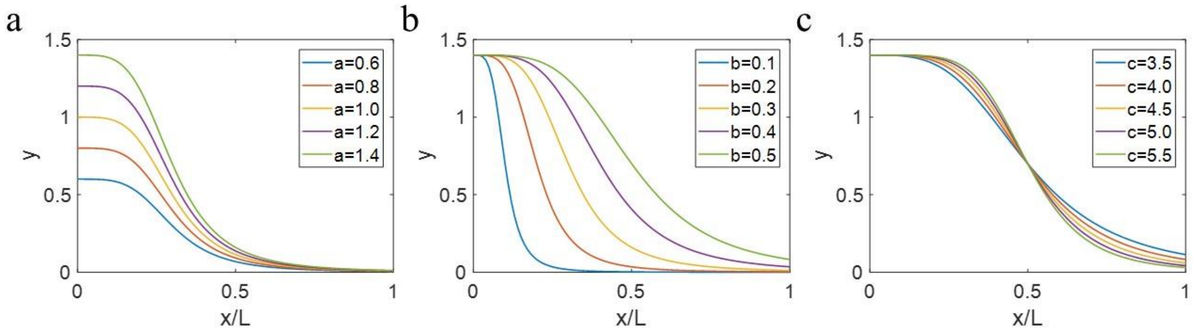

Figure S1. The interpretation of how the curve of Hill equation (equation 1) changes with each parameter.
(a) The amplitude of the Hill equation curve increases as parameter a increases while fixing b and c. **(b)** The curve gets steeper as b decreases while fixing a and c. **(c)** The curve passes through a fixed point at $(b, \frac{a}{2})$, and the slope at the fixed point increases as c decrease.
